# Development and validation of a TRP-related gene signature for overall survival prediction in lung adenocarcinoma

**DOI:** 10.3389/fgene.2022.905650

**Published:** 2022-09-15

**Authors:** Min He, Gujie Wu, Ziheng Wang, Kuan Ren, Zheng Yang, Qun Xue

**Affiliations:** ^1^ Medical College of Nantong University, Nantong, Jiangsu, China; ^2^ Research Center of Clinical Medicine, Affiliated Hospital of Nantong University, Nantong, Jiangsu, China; ^3^ Cardiothoracic Surgery Department, Affiliated Hospital of Nantong University, Nantong, Jiangsu, China

**Keywords:** lung adenocarcinoma, prognosis, immune, TRP-related gene, gene signature

## Abstract

The transient receptor potential (TRP) channel is a type of channel protein widely distributed in peripheral and central nervous systems. Genes encoding TRP can be regulated by natural aromatic substances and serve as a therapeutic target for many diseases. However, the role of TRP-related genes in lung adenocarcinoma (LUAD) remains unclear. In this study, we used data from TCGA to screen and identify 17 TRP-related genes that are differentially expressed between LUAD and normal lung tissues. Based on these differentially expressed genes (DEGs), we classified all patients with LUAD into two subtypes. Significant differences in prognosis, clinical features, and immune cell infiltration characteristics were observed between the two subtypes. Subsequently, a prognostic signature with 12 genes was established by applying the least absolute shrinkage and selection operator (LASSO) Cox regression method, and all patients with LUAD were classified into low- and high-risk groups. Patients with LUAD in the low-risk group had a significantly longer survival time than those in the high-risk group (*p* < 0.001), which was confirmed by LUAD data from the GSE72094 and GSE68571 validation datasets. Combined with clinical characteristics, the risk score was found to be an independent predictor of overall survival (OS) in patients with LUAD. Additionally, patients with high TRP scores exhibited poorer clinical characteristics and immune status while showing a sensitive response to chemotherapeutic agents. In conclusion, the TRP score is a promising biomarker for determining the prognosis, molecular subtype, tumor microenvironment, and guiding personalized treatment in patients with LUAD.

## Introduction

Lung cancer is one of the most common causes of cancer-related deaths worldwide. Primary lung cancer is caused by exposure to smoking, ionizing radiation, and environmental pollution. Non-small cell lung cancer (NSCLC) accounts for the vast majority of lung cancer cases, among which lung adenocarcinoma (LUAD) is the most common histological subtype of lung cancer (Denisenko et al., 2018; Bade and Dela Cruz, 2020; Zhang et al., 2020)_._ Despite recent developments in targeted therapy and immunotherapy in the treatment of lung cancer, which have improved patient prognosis, the 5-year survival rate remains below 20% (Hirsch et al., 2017; Greenawalt et al., 2019). Given the limitations of LUAD treatment, new therapeutic targets are needed to improve clinical outcomes; therefore, there is an urgent need to establish reliable and novel prognostic models to make targeted therapy more feasible.

Members of the TRP channel family are potential biomarkers and drug targets in tumor therapy (Xing et al., 2021). Recent studies have identified the TRP channel interactome as a new therapeutic target for breast cancer and have shown that it promotes proliferation and poor prognosis in esophageal squamous cell carcinoma (María Paz et al., 2021; Wang et al., 2021). In addition, TRP-related genes are associated with tumor progression in adenocarcinoma of the prostate (Di Donato et al., 2021) and colon (Liu et al., 2022) and urothelial carcinoma of the bladder (Shapovalov et al., 2016). Therefore, uncovering the role of TRP-related genes in the prognosis of LUAD would be of great significance.

In this study, we performed a systematic expression analysis of TRP-related genes between the normal lung and LUAD tissues and explored the prognostic value of these genes. A 12-gene TRP-related prognostic signature was developed by analyzing RNA-seq data from patients with LUAD in TCGA database and corresponding clinical information. Subsequently, further validation was obtained by analyzing data from the GSE72094 and GSE68571 cohorts. Finally, we analyzed the correlation between TRP-related prognostic signatures and the tumor immune microenvironment. Thus, our data may provide additional evidence for prognostic biomarkers and therapeutic targets in LUAD.

## Materials and methods

### Data collection

The transcriptome data in the FPKM format and corresponding clinical information of 58 para-cancerous samples and 497 LUAD samples were collected from TCGA database (https://portal.gdc.cancer.gov/). The expression data for GSE72094 and GSE68571 cohorts, as well as the clinical data, were downloaded from the GEO (https://www.ncbi.nlm.nih.gov/geo/) database as the validation set for the risk model. TRP-related genes were derived from the REACTOME_TRP_CHANNELS pathway in the MsigDB database and inflammatory mediator regulation of the TRP channel pathway in the KEGG database, and those genes with duplicate values were removed.

### Differential expression analysis

Differential expression analysis was performed on LUAD samples and normal samples from TCGA using the *limma* R package. The Benjamini–Hochberg (FDR) corrected *p*-value adj. *p*-value < 0.05 and |log2 FC| > 0.585 were used as thresholds to screen for DEGs. The same method was used to perform differential expression analysis between tumor subtypes and to screen for genes with significant differences in their expression.

### Clustering analysis

Consistent clustering analysis was performed on LUAD samples using the *ConsensusClusterPlus* R package. The Euclidean method was used to calculate the clustering distance (the clustering method was k-means), and 100 replications were performed to ensure the stability of the classification. KM survival curves were plotted for patients with subtypes using the *survival* R package, and the significance of prognostic differences between subtypes was determined using the log-rank test.

### Construction of the prognostic risk model and survival difference analysis

One-way Cox regression analysis was performed to screen genes associated with prognosis (OS) based on DEGs among tumor subtypes (*p* < 0.01). Finally, LASSO regression was used to further screen out key prognosis-related genes and construct a multifactorial regression prognostic model. Tumor samples were classified into high- and low-risk groups using the median risk score as the threshold point, and survival curves for prognostic analysis were generated by the Kaplan–Meier method, and the significance of differences was determined using log-rank tests. ROC (receiver operating characteristic) curves were then plotted using the *timeROC* R package to evaluate the prediction of scoring by the perturbation scoring model. The risk score was calculated using the following formula: $\mathrm{Risk}\;\mathrm{score=}\beta_{1}*X_{1}+\beta_{2}*X_{2}+\ldots +\beta_{i}*X_{i}$ ( $\beta_{i}$ : weighting factor for each gene; $X_{i}$ : [log2FPKM] for each gene). The risk value of the model is the sum of each candidate gene expression value multiplied by its weight.

### Analysis of immune score and immune infiltration

The CIBERSORT algorithm is a method to characterize complex tissues based on their gene expression profiles. A leukocyte signature gene matrix of 547 genes, LM22, was used to distinguish between 22 immune cell types, including myeloid subpopulations, natural killer (NK) cells, plasma cells, naive and memory B cells, and seven T-cell types. CIBERSORT combined with the LM22 signature matrix was used to estimate the proportion of 22 cell phenotypes in samples, with the sum of the proportions of all immune cell types in each sample equal to 1.

Immune checkpoints refer to a series of molecules expressed in immune cells that regulate the level of immune activation and play an important role in the occurrence of autoimmune diseases. This study aimed to explore the correlation between immune checkpoint gene expression and TRPRS.

### Drug sensitivity analysis

The sensitivity of 138 drugs in the GDSC database (IC_50_ values) was predicted using the *pRRophetic* R package, combined with the expression data of model genes, and the sensitivity of patients with LUAD to drug treatment was assessed by IC_50_ values. The differences in IC_50_ values between high- and low-risk groups were also compared by the Wilcoxon test, and drugs with significant differences between the two groups were screened.

### Mutation display

Waterfall plots were plotted using the *maftools* R package to demonstrate mutations in LUAD samples with mutation frequencies in the top 20 genes and differences in mutations in high- and low-risk groups. The analysis of copy number variations in LUAD samples from TCGA was performed using GISTIC2, and these variations were mapped to highlight CNV differences between the high- and low-risk groups.

### Statistical analysis

The Wilcoxon test was used to compare differences between two sample groups, and the Kruskal–Wallis test was used to compare differences between multiple sample groups, where ns indicates *p* > 0.05, * indicates *p* ≤0.05, ** indicates *p* ≤0.01, *** indicates *p* ≤0.001, and **** indicates *p* ≤0.0001.

## Results

### Analysis of the dysregulated expression of transient receptor potential-related genes in tumors

**FIGURE 1:**
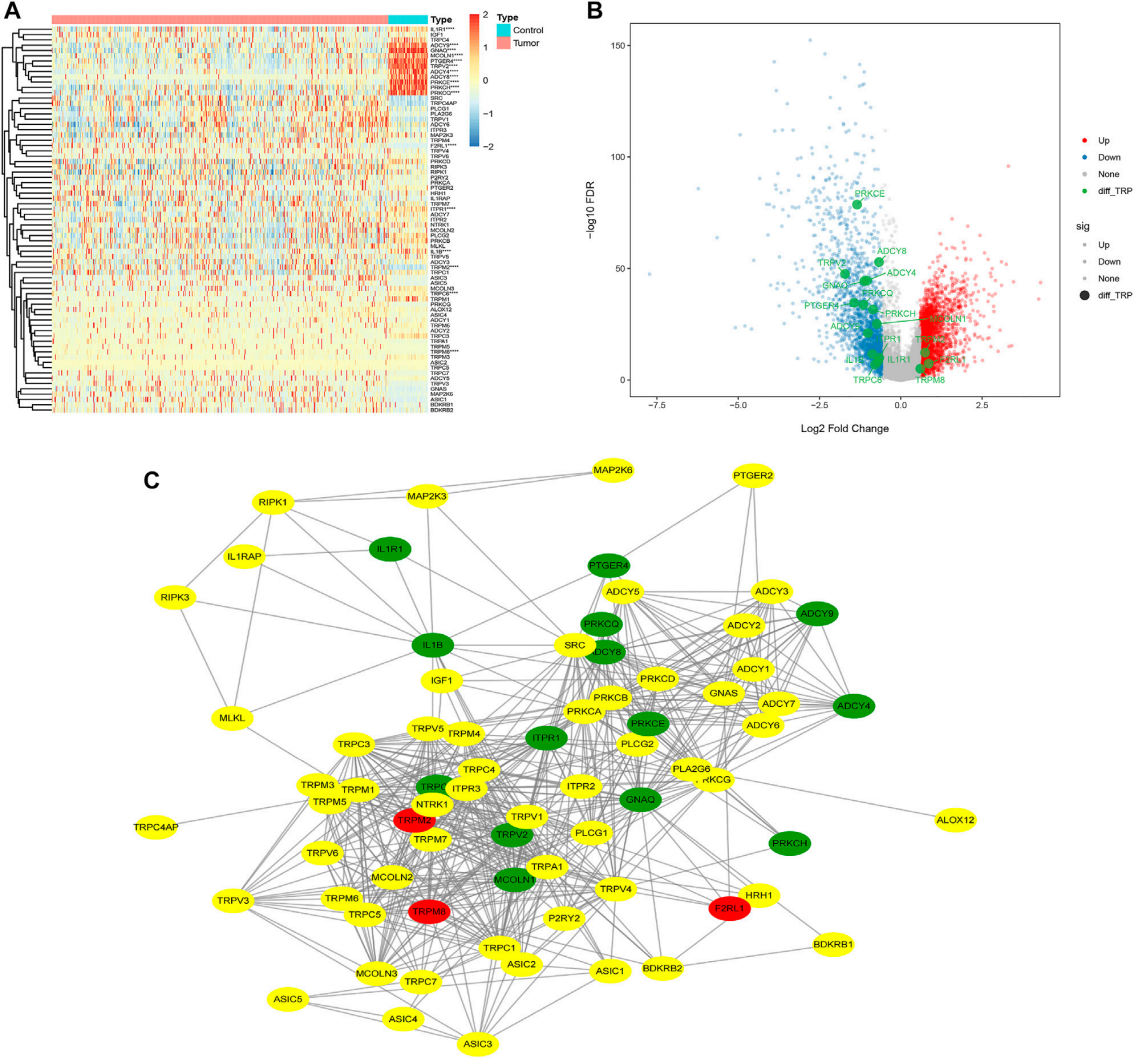
Differential heatmap of 73 TRP‐related genes **(A)**; volcano map shows the 17 of the most significantly DEGs **(B)**; Protein‐protein Interaction (PPI) of TRP‐related Genes **(C)**.

**FIGURE 2:**
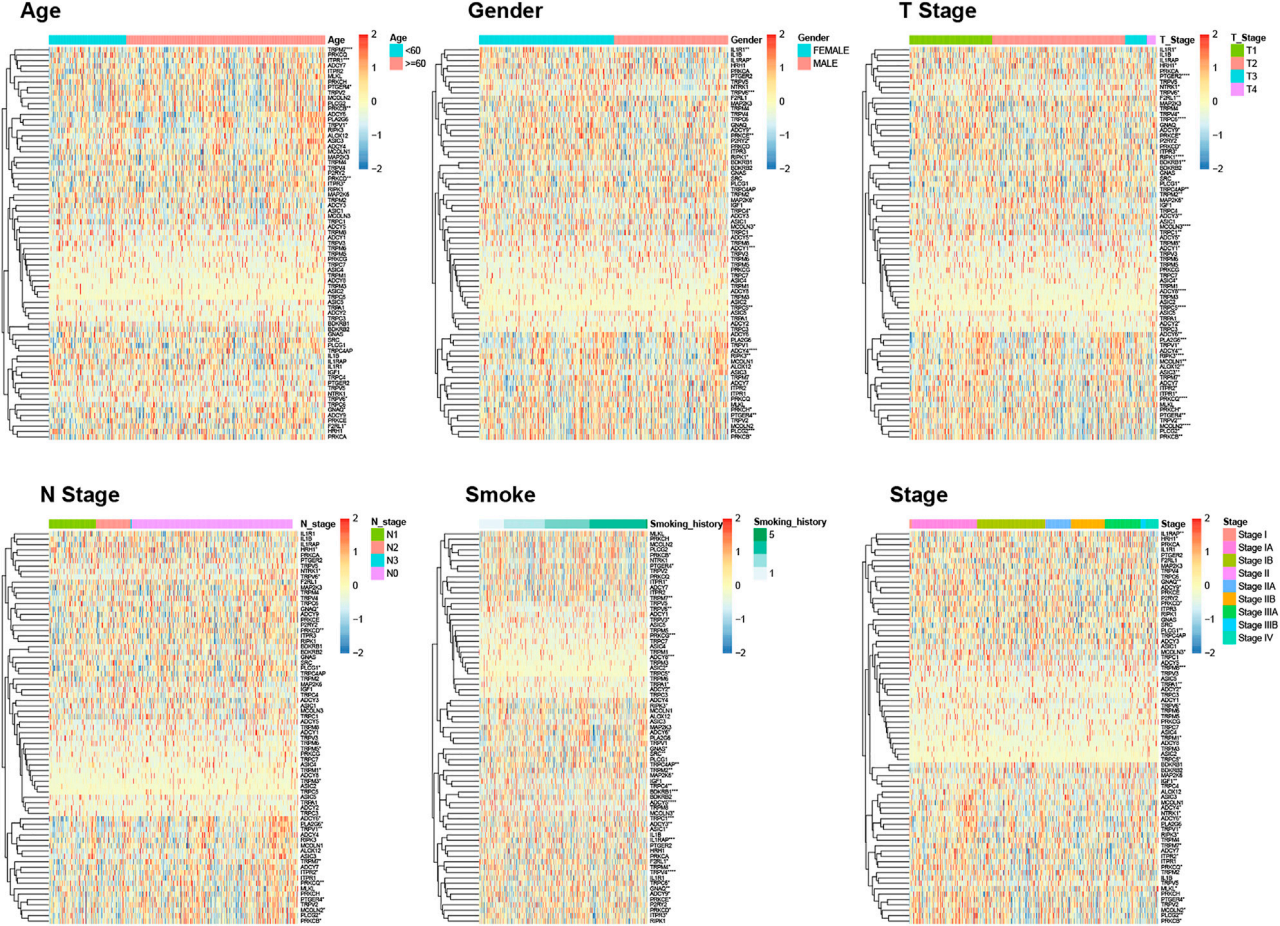
Differential heatmap of TRP-related genes in different clinical subgroups.

First, we obtained 4,363 DEGs, including 73 TRP-related genes, using differential expression analysis of tumor samples (N = 497) and normal samples (N = 58) (Figure 1A, *p* < 0.05). Then, we selected 17 of the most significantly DEGs to plot the gene volcanoes (Figure 1B, *p* < 0.001). To better understand TRP-related gene interactions, we used the STRING database to obtain TRP-related protein–protein interaction (PPI) networks, with red representing upregulated genes and green representing downregulated genes (Figure 1C). Subsequently, we analyzed the differences in the expression of TRP-related genes in different clinical subgroups according to the grouping of the clinical information of the samples (Figure 2).

### Tumor classification based on the transient receptor potential-related genes

**FIGURE 3:**
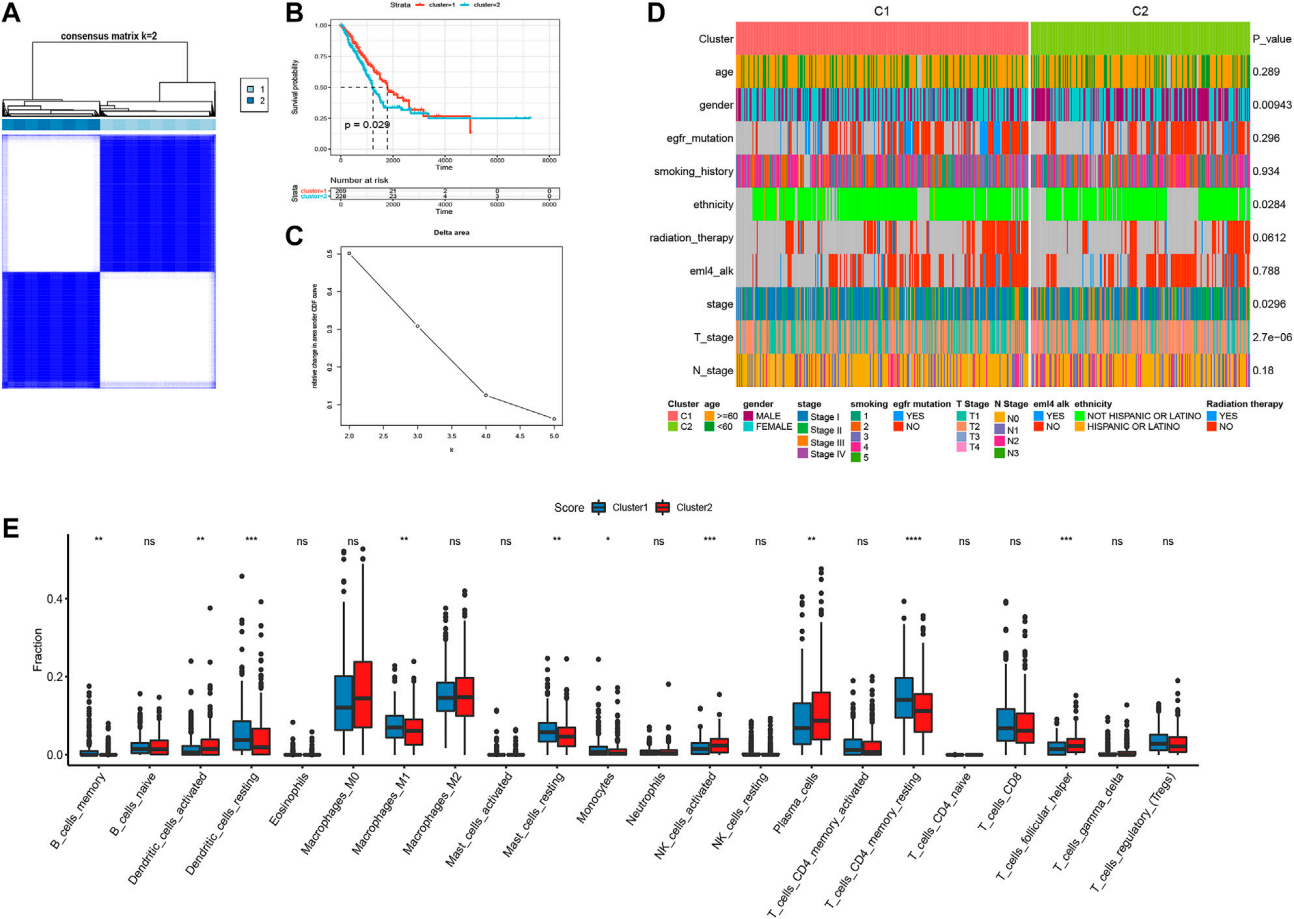
Consistent clustering result of tumor samples **(A)**; Survival curves and CDF curves of the two subtypes were obtained according to the clustering results **(B,C)**; Heat map of TRP subtypes and clinical features **(D)**; 22 immune cell infiltration differences between subtypes **(E)**. *p < 0.05, **p < 0.01, ***p < 0.001, ****p < 0.0001.

Based on all TRP-related genes, 497 tumor samples were classified into two different subtypes using consistent clustering: cluster1 (N = 269) and cluster2 (N = 228) (Figure 3A). The results from the Kaplan–Meier plot showed the significant differences in the survival probability and recurrence rate among these two subtypes (Figure 3B, *p* = 0.029). We further analyzed the CDF delta area curve and found that the area under the CDF curve tended to be stable after two clusters (Figure 3C). Heat maps of TRP subtypes and clinical features were generated from the clustering results, and statistical tests were performed to calculate the significance of the correlation between the results of subtype grouping and those of clinical feature grouping. We found that sex, ethnicity, T stage, and tumor stage were significantly associated with TRP subtypes (Figure 3D). Finally, we clustered the TCGA-LUAD dataset, calculated the proportion of 22 types of immune cells between subtypes, and found significant differences in immune cell infiltration between the two subtypes (Figure 3E).

### Transient receptor potential-related prognostic signature construction

**FIGURE 4:**
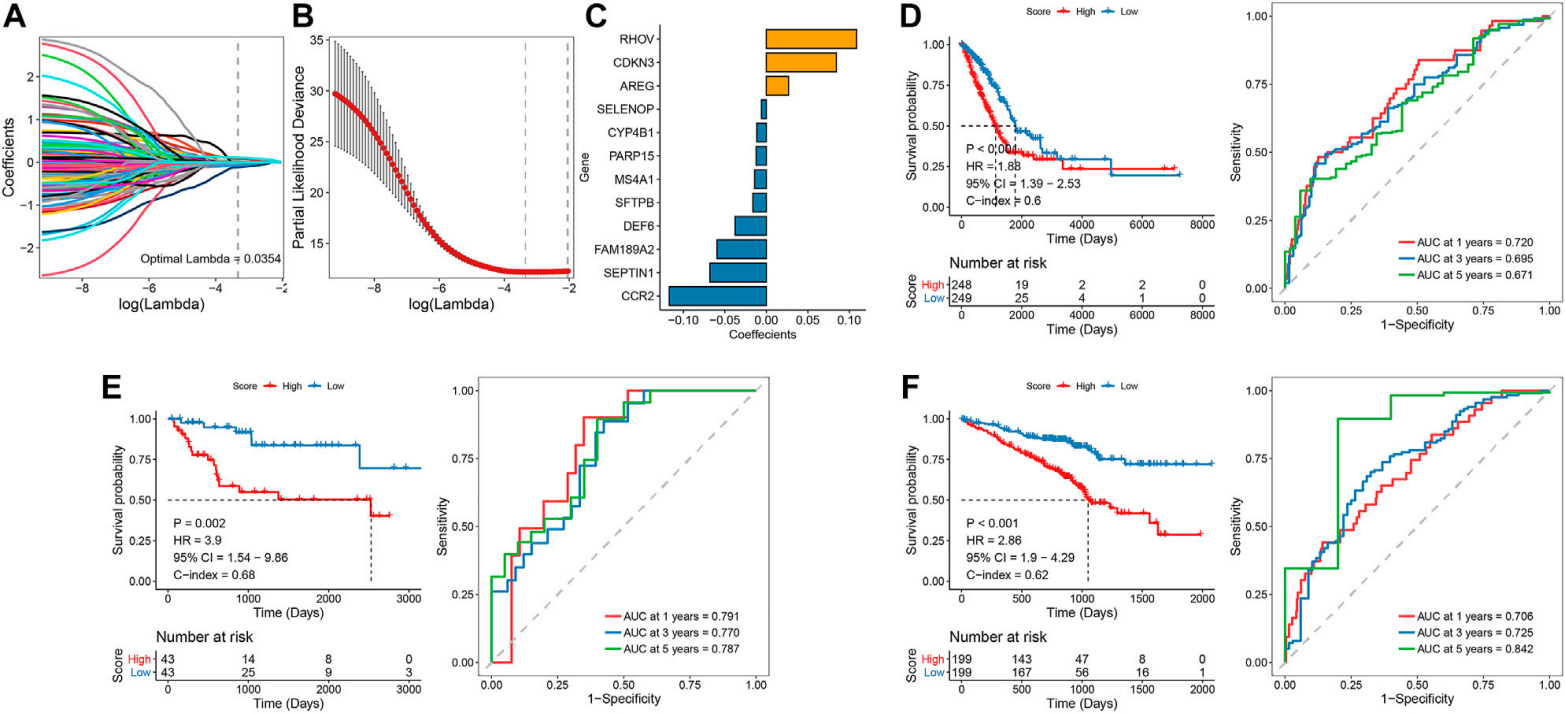
LASSO linear regression and univariate Cox analysis screened 12 feature genes associated with prognosis **(A-C)**; Survival curve and ROC curve between the high-risk and low-risk groups, verified by E and F validation sets **(D-F)**.

We performed univariate Cox screening of prognosis-related genes using DEGs among TRP subtypes, resulting in 158 prognostic factors. The univariate Cox results were then further downscaled using LASSO linear regression to screen 12 prognostic-related signatures (Figures 4A–C). The samples from the TCGA LUAD training set were divided into two groups of high and low risk using the median risk score as the threshold. As shown in Figure 4D, there was a significant survival difference between high- and low-risk groups. The ROC curves for the prognostic features had AUC values of 0.720/0.695/0.671 at 1/3/5 years. Subsequently, we validated and confirmed the validity of the signature in the GSE72094 and GSE68571 validation datasets (Figures 4E, F).

### Risk score and correlations with clinicopathological characteristics

**FIGURE 5:**
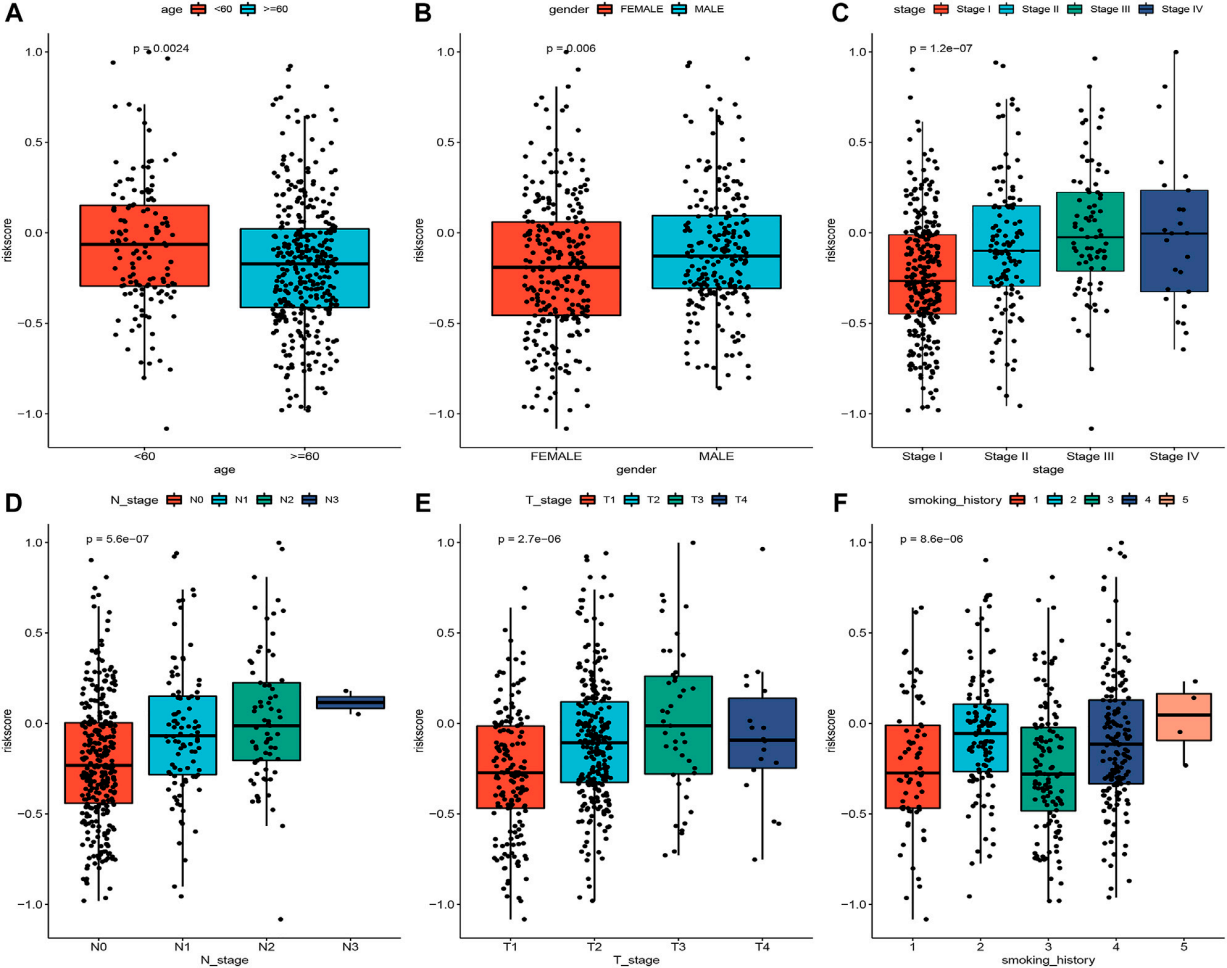
Correlation between risk scores and different clinical characteristics **(A–F)**.

**FIGURE 6:**
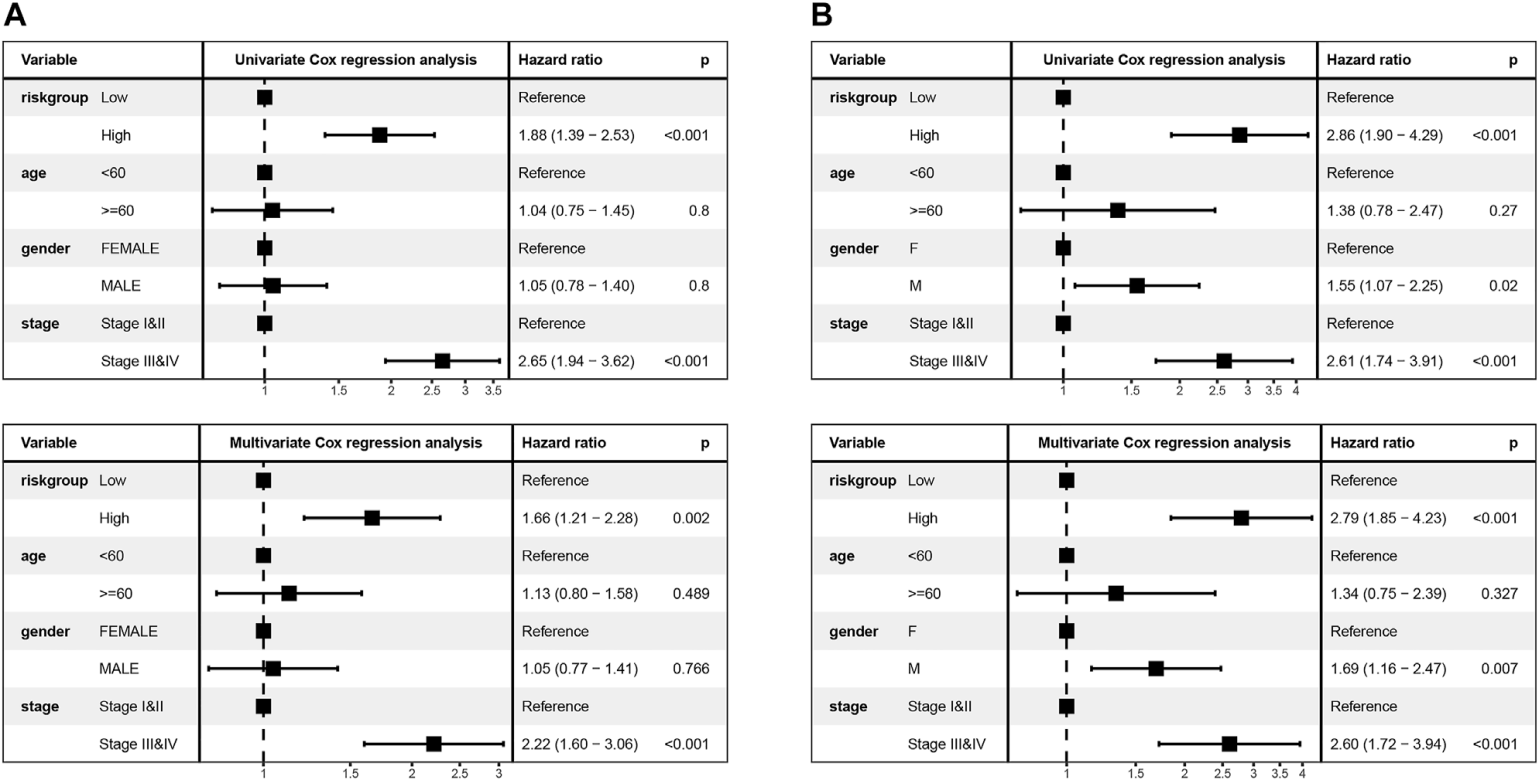
Univariate and multivariate Cox regression analyses were performed to assess the risk score and verified by validation set B **(A,B)**.

**FIGURE 7:**
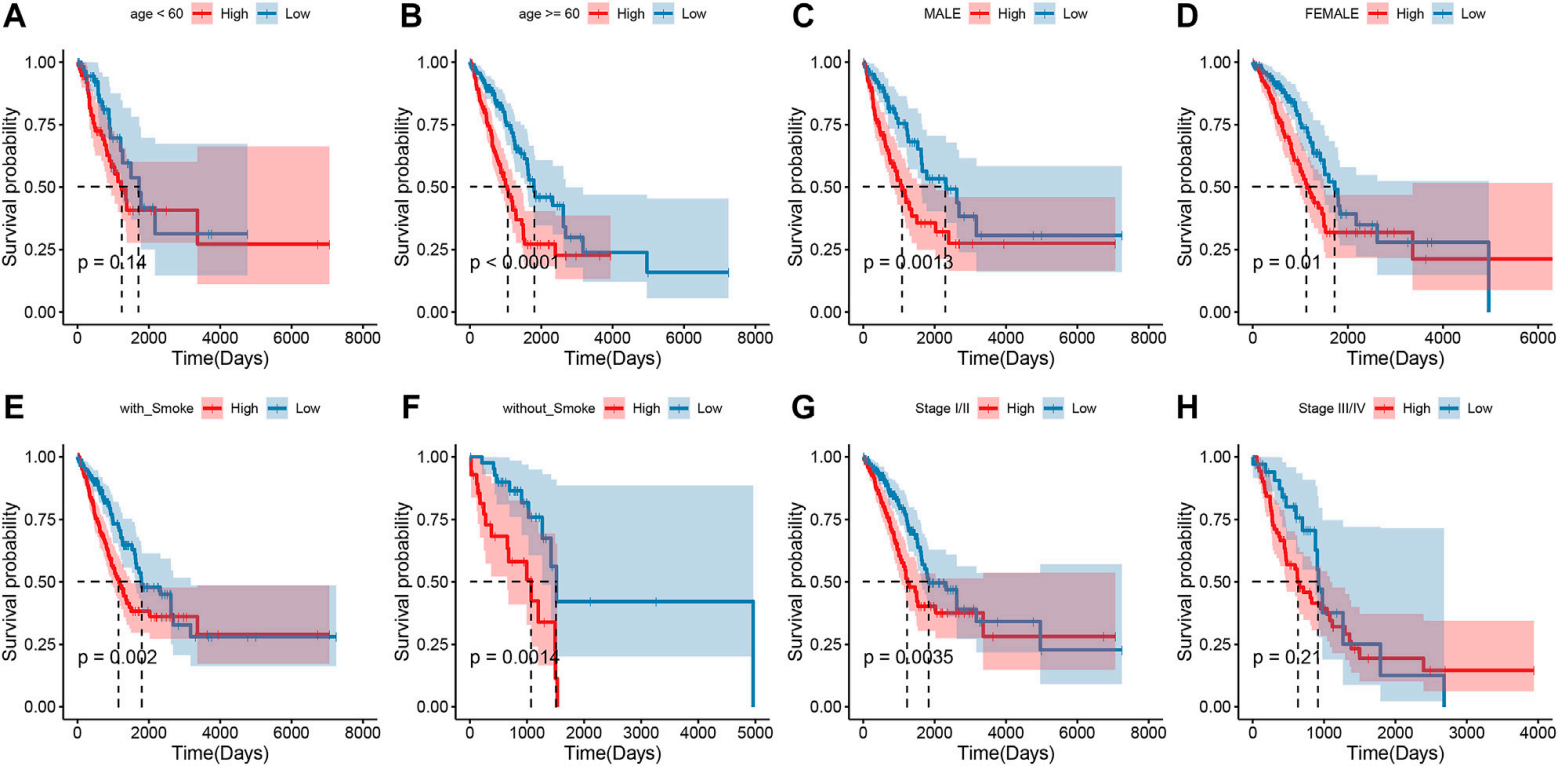
Survival outcomes of patients with different clinical characteristics in the high and low groups **(A–H)**.

To explore the association of risk scores with different clinical characteristics, we performed a subgroup analysis of different clinical characteristics and found significant differences in risk scores with respect to age (*p* = 0.0024), sex (*p* = 0.006), TNM stage (*p* = 1.2e-07), N stage (*p* = 5.6e-07), T stage (*p* = 2.7e-06), and smoking history (*p* = 8.6e-06) (Figures 5A–F). We used univariate and multivariate Cox regression analyses to assess whether the risk score could be used as an independent prognostic factor. The results of TCGA training set validation are shown in Figure 6A. Risk scores and tumor stage were independent factors for poor survival in patients with LUAD. This result was further verified by univariate and multivariate analyses of the GSE72094 validation set (Figure 6B). We also performed chi-squared tests for clinical characteristics such as age, gender, stage, and smoking in control and tumor groups, which showed no significant differences (Supplementary Table S1). In addition, the risk score was a good predictor of patient prognosis in groups with different clinical characteristics (Figures 7A–H).

### Risk score and correlations with the tumor microenvironment

**FIGURE 8:**
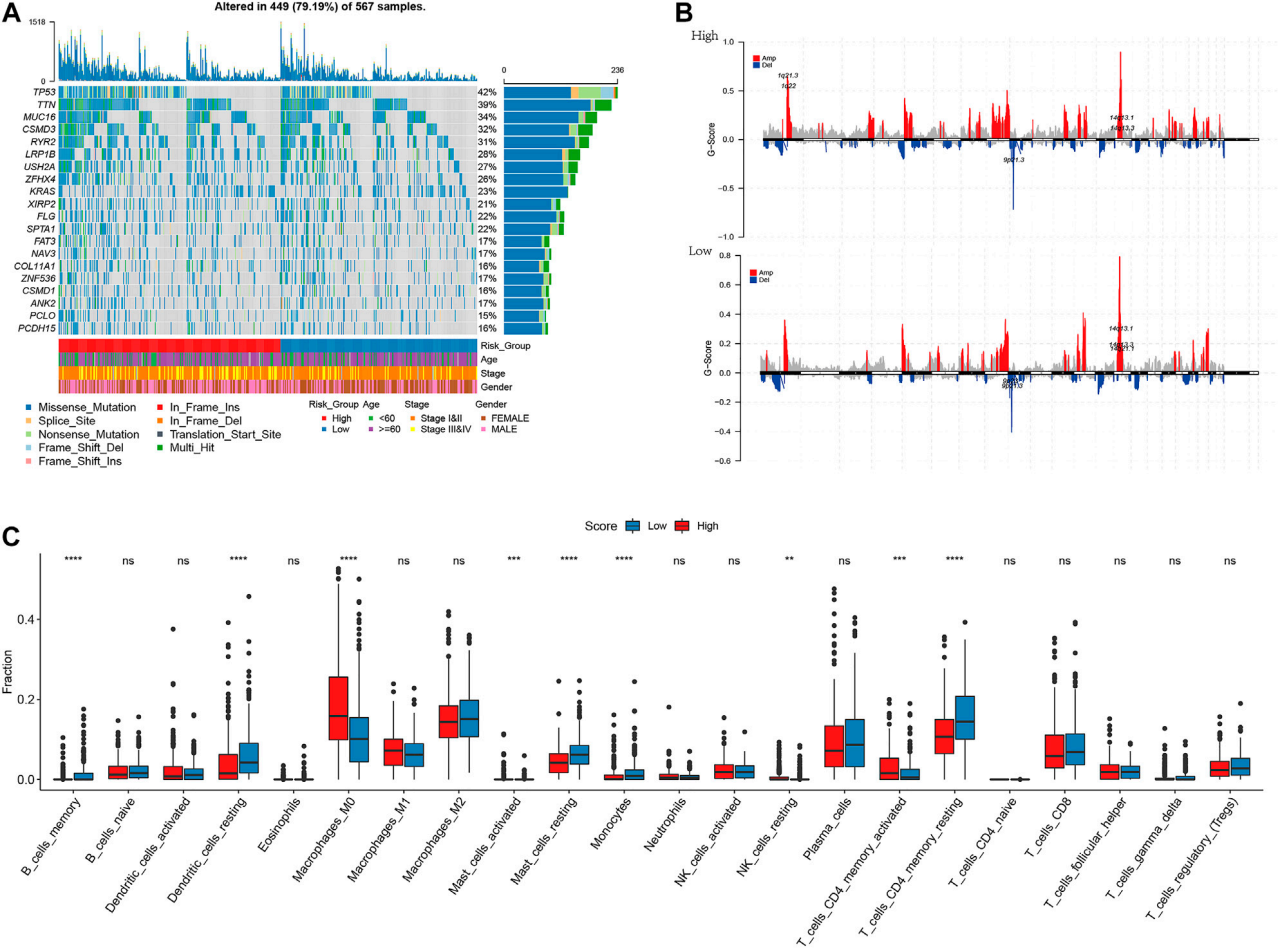
I plotted the waterfall and selected the top 20 genes with the highest frequency of mutations **(A)**; Amplification and deletion of chromosomes in the high-low risk groups **(B)**; Differential results of immune infiltration in high-low risk groups **(C)**. *p < 0.05, **p < 0.01, ***p < 0.001, ****p < 0.0001.

Mutations in genes can promote, cause, or orchestrate the malignant progression of tumors, and the study of mutations at the genomic level is important for the development of targeted cancer drugs and novel cancer therapies. To demonstrate the distribution of somatic variants between high- and low-risk groups and to demonstrate the distribution of mutations between samples with different clinical characteristics, the top 20 genes with the highest mutation frequencies were selected for the waterfall plot, as shown in Figure 8A Supplementary Table S2. Because the prognosis of patients in the high-risk group was worse than that of patients in the low-risk group, we speculated whether the risk degree is related to the mutations of some common cancer-promoting genes. Therefore, we further analyzed the gene mutations between high- and low-risk groups. We found a higher frequency of mutations in cancer-promoting genes, such as *TP53*, *MUC16*, and *TTN*, in the high-risk group. Therefore, we demonstrated a correlation between the expression of the degree of risk and the mutation of cancer-promoting genes. Additionally, we compared whether high- and low-risk groups had different levels of amplification and deletion. In high- and low-risk groups, we counted the number of amplifications and deletions of 22 groups of chromosomes. As shown in Figure 8B and Supplementary Table S3, there was a large degree of amplification and deletion in the high-risk group. To explore the correlation between high- and low-risk groups and the tumor microenvironment, we calculated the difference in immune infiltration between high- and low-risk groups based on the TCGA-LUAD dataset. As shown in Figure 8C, we found significant differences between the low- and high-risk groups in nine immune cell types (memory B cells, resting dendritic cells, M0 macrophages, activated mast cells, resting mast cells, monocytes, resting NK cells, memory-activated CD4 T cells, and memory-resting CD4 CT cells). The low-risk group had more immune cell infiltration than the high-risk group.

### Risk score and correlations with chemotherapy response

**FIGURE 9:**
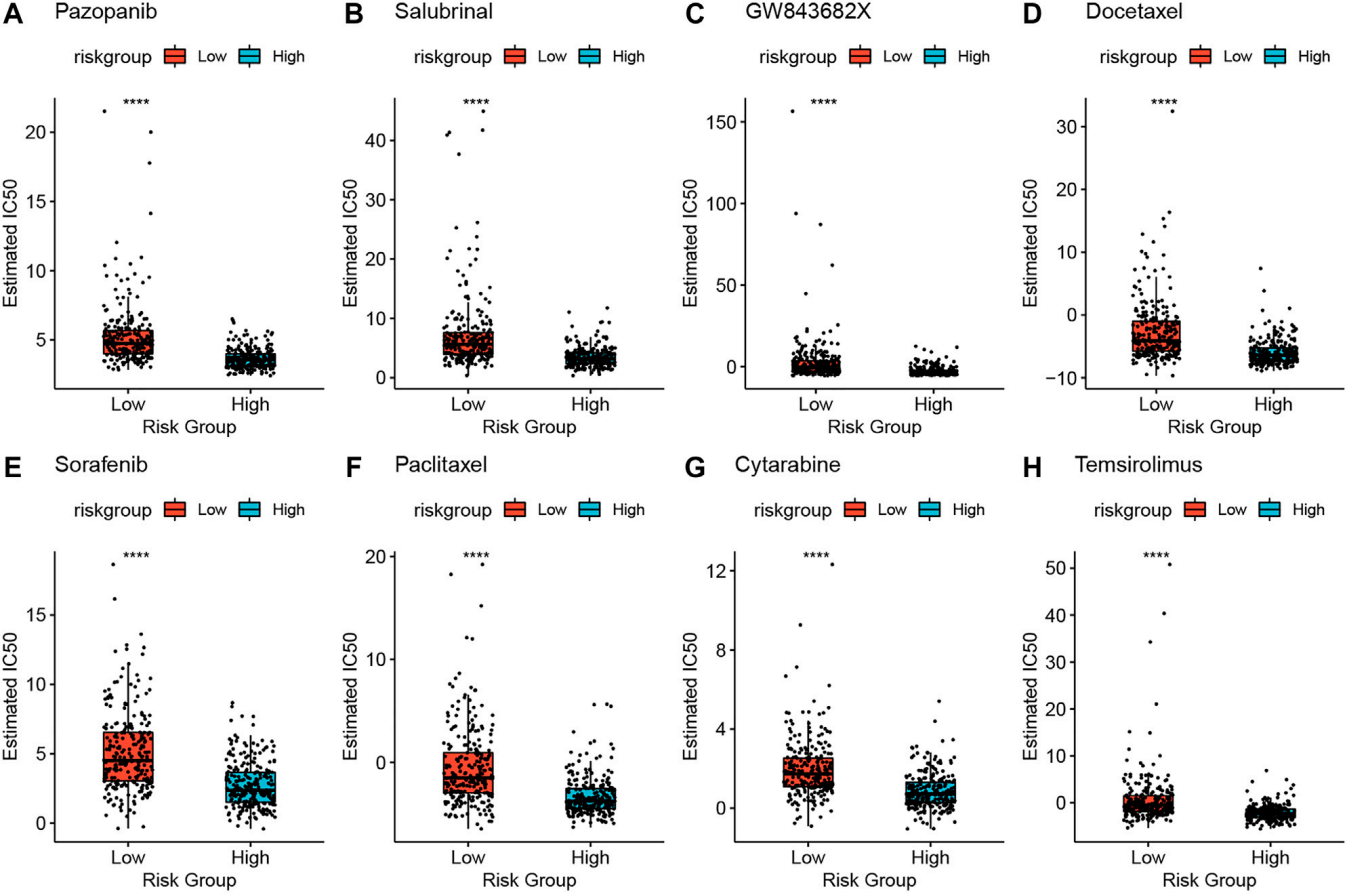
High-risk and low-risk groups respond differently to the eight drugs **(A–H)**. *p < 0.05, **p < 0.01, ***p < 0.001, ****p < 0.0001.

To screen for antineoplastic drugs, we predicted the sensitivity (IC_50_ values) of 138 drugs in the GDSC database based on the expression profiles of all the characterized genes in our model and screened 53 LUAD-associated drugs (Table 1). Then, we found that response to 41 drugs differed significantly between high- and low-risk groups, and the top 8 drugs (pazopanib, salubrinal, GW843682X, docetaxel, sorafenib, paclitaxel, cytarabine, and temsirolimus) with the most significant differences in their responses are shown in Figure 9.

**TABLE 1:** 53 LUAD-associated drugs.

y	Group1	Group2	*p*	p.adj	p.format	p.signif	Method
Pazopanib	Low	High	1.02E-35	1.00E-35	< 2e-16	****	Wilcoxon
Salubrinal	Low	High	3.75E-32	3.70E-32	< 2e-16	****	Wilcoxon
GW843682X	Low	High	1.36E-28	1.40E-28	< 2e-16	****	Wilcoxon
Docetaxel	Low	High	8.41E-28	8.40E-28	< 2e-16	****	Wilcoxon
Sorafenib	Low	High	6.42E-26	6.40E-26	< 2e-16	****	Wilcoxon
Paclitaxel	Low	High	2.72E-25	2.70E-25	< 2e-16	****	Wilcoxon
Cytarabine	Low	High	6.83E-25	6.80E-25	< 2e-16	****	Wilcoxon
Temsirolimus	Low	High	2.52E-24	2.50E-24	< 2e-16	****	Wilcoxon
Bortezomib	Low	High	2.58E-21	2.60E-21	< 2e-16	****	Wilcoxon
CMK	Low	High	3.20E-21	3.20E-21	< 2e-16	****	Wilcoxon
Elesclomol	Low	High	9.21E-19	9.20E-19	< 2e-16	****	Wilcoxon
Pyrimethamine	Low	High	3.69E-18	3.70E-18	< 2e-16	****	Wilcoxon
CCT007093	Low	High	3.31E-17	3.30E-17	< 2e-16	****	Wilcoxon
AZD8055	Low	High	2.10E-16	2.10E-16	< 2e-16	****	Wilcoxon
Vorinostat	Low	High	2.16E-16	2.20E-16	< 2e-16	****	Wilcoxon
Vinblastine	Low	High	8.49E-16	8.50E-16	8.50E-16	****	Wilcoxon
Shikonin	Low	High	8.82E-16	8.80E-16	8.80E-16	****	Wilcoxon
Rapamycin	Low	High	6.04E-15	6.00E-15	6.00E-15	****	Wilcoxon
Erlotinib	Low	High	4.78E-14	4.80E-14	4.80E-14	****	Wilcoxon
Methotrexate	Low	High	8.87E-14	8.90E-14	8.90E-14	****	Wilcoxon
DMOG	Low	High	7.48E-13	7.50E-13	7.50E-13	****	Wilcoxon
Axitinib	Low	High	1.48E-12	1.50E-12	1.50E-12	****	Wilcoxon
Lapatinib	Low	High	2.81E-12	2.80E-12	2.80E-12	****	Wilcoxon
Tipifarnib	Low	High	9.08E-12	9.10E-12	9.10E-12	****	Wilcoxon
Cisplatin	Low	High	2.45E-11	2.40E-11	2.40E-11	****	Wilcoxon
SB590885	Low	High	2.19E-08	2.20E-08	2.20E-08	****	Wilcoxon
Bexarotene	Low	High	1.84E-07	1.80E-07	1.80E-07	****	Wilcoxon
AZ628	Low	High	8.69E-06	8.70E-06	8.70E-06	****	Wilcoxon
Etoposide	Low	High	1.95E-05	2.00E-05	2.00E-05	****	Wilcoxon
Lenalidomide	Low	High	3.07E-05	3.10E-05	3.10E-05	****	Wilcoxon
Embelin	Low	High	3.24E-05	3.20E-05	3.20E-05	****	Wilcoxon
Imatinib	Low	High	5.76E-05	5.80E-05	5.80E-05	****	Wilcoxon
Thapsigargin	Low	High	8.88E-05	8.90E-05	8.90E-05	****	Wilcoxon
Camptothecin	Low	High	0.000224914851455118	0.00022	0.00022	***	Wilcoxon
Gemcitabine	Low	High	0.000556403606421614	0.00056	0.00056	***	Wilcoxon
FH535	Low	High	0.000578246872199532	0.00058	0.00058	***	Wilcoxon
AZD6482	Low	High	0.0101272818535892	0.01	0.01013	*	Wilcoxon
Nilotinib	Low	High	0.0109494621404446	0.011	0.01095	*	Wilcoxon
Parthenolide	Low	High	0.0105378894590561	0.011	0.01054	*	Wilcoxon
Vinorelbine	Low	High	0.0290182377811531	0.029	0.02902	*	Wilcoxon
Bleomycin	Low	High	0.029126211188871	0.029	0.02913	*	Wilcoxon
Sunitinib	Low	High	0.068383120007987	0.068	0.06838	ns	Wilcoxon
GSK269962A	Low	High	0.0848611578629623	0.085	0.08486	ns	Wilcoxon
Dasatinib	Low	High	0.0871069712669459	0.087	0.08711	ns	Wilcoxon
Doxorubicin	Low	High	0.318323080639613	0.32	0.31832	ns	Wilcoxon
AS601245	Low	High	0.323173430084827	0.32	0.32317	ns	Wilcoxon
AZD7762	Low	High	0.363853871726053	0.36	0.36385	ns	Wilcoxon
Bicalutamide	Low	High	0.544354106852985	0.54	0.54435	ns	Wilcoxon
Gefitinib	Low	High	0.559528107176579	0.56	0.55953	ns	Wilcoxon
Midostaurin	Low	High	0.605884604352698	0.61	0.60588	ns	Wilcoxon
Bosutinib	Low	High	0.856310661540995	0.86	0.85631	ns	Wilcoxon
QS11	Low	High	0.881705656416548	0.88	0.88171	ns	Wilcoxon
Cyclopamine	Low	High	0.896062326936675	0.9	0.89606	ns	Wilcoxon

## Discussion

Transient receptor potential (TRP) channels are a versatile family of ion channels. Within the TRP ion channel family, TRPV1 primarily mediates pain and burning induced by spicy compounds in the somatosensory system (Steinritz et al., 2018). TRP channels play an important role not only in mediating pain but also in the cell cycle, often by regulating gene transcription and affecting other cellular processes such as proliferation, apoptosis, or cell motility (Shapovalov et al., 2016; Yu et al., 2016). Previous studies have shown that the TRP ion channel family is associated with the progression of several cancers. For example, the increased expression of TRPM7 is associated with poor prognosis and metastasis in nasopharyngeal carcinoma. TRPC1, TRPC5/6, TRPM4, TRPM7/8, TRPV1/2, TRPV4, and TRPV6 are strongly associated with progression and could be new therapeutic targets for breast invasive carcinoma (Chen et al., 2015; Saldías et al., 2021). In addition, several studies have identified the TRP ion channel family genes as promising predictors of prognosis and immunotherapeutic efficacy in patients with cancer through pan-cancer analysis (Pan et al., 2022; Wu et al., 2022). However, the role of the TRP ion channel family in LUAD remains elusive. Therefore, we searched for TRP-related genes through the TRP pathway and performed a clustering analysis of patients with LAUD. Then, to investigate the link between patient prognosis and the TRP pathway, we built a survival prediction model based on TRP-related subtypes.

In this study, we downloaded expression profile data and mutation data from TCGA database and analyzed them for differential expression. After screening for DEGs, we performed clustering analysis and determined the differential relationships between subtypes and prognosis, clinical features, and immune infiltration. The focus of this study was to screen out prognostic genes and construct a TRP prognostic stratification scoring system. We downloaded and screened TRP-related crossover genes from MsigDB and KEGG databases, screened 12 prognosis-related signatures, and used the median gene expression as the cutoff value for high- and low-risk groups. The expression data and clinical data were downloaded from the GEO database as the validation set for the risk model, and the stability of the model validity was tested by evaluating and validating training and validation sets. Some of these 12 signatures have been confirmed to be closely related to the occurrence and development of lung cancer. For example, induction of AREG expression sensitizes lung cancer cells to EGFR TKI and increases the tumorigenic dependence of non-small cell lung cancer on the AREG-induced EGFR signaling pathway, thus enhancing the progression of NSCLC (Tu et al., 2018). High CCR2 expression is associated with a poor prognosis of various cancers, while inhibition of CCR2 expression may enhance the inhibitory effect of PD-1 on tumors. Simultaneously, Yi et al. discovered that CCR2 expression was connected with prognosis, favorably correlated with survival rate and prognosis of the M type, negatively correlated with the prognosis of T and N types, and correlated with immune cell infiltration of different malignancies (An et al., 2017). Functional CDKN3, but not dominant-negative CDKN3 mutants, is overexpressed in LUAD, and overexpression of major CDKN3 transcripts is associated with poor survival in patients with LUAD (Fan et al., 2015). RHOV is expressed in lung cancer cell lines and is upregulated in most of the lung tumor cases studied (Shepelev and Korobko, 2013). The overexpression of RHOV in LUAD promotes the progression of LUAD and EGFR-TKI resistance, which may be related to the activation of the AKT/ERK pathway (Chen et al., 2021). RHOV plays a key role in LUAD metastasis and may provide a biomarker for the prognosis and treatment of LUAD (Zhang et al., 2021). Correlations between different clinical characteristics and model scores were explored based on the grouping of clinical characteristics and risk values for each sample in the model. Based on univariate and multivariate Cox regression analyses, we found that risk groups can be used as independent risk factors. In difference analysis, chi-squared tests were performed on the clinical traits such as age, gender, stage, and smoking in the control group and tumor group, and no significant differences were found. The risk score also constructs the survival curve of clinical characteristics, which is clearly important in guiding clinical outcomes. We found that the high- and low-risk groups not only possessed higher mutations and CNV alterations but also predicted worse prognosis, clinical characteristics, and the tumor microenvironment in patients with LUAD. In addition to that, there were significant differences in the frequencies of mutations between high- and low-risk groups for COL11A1, CSMD3, FAT3, LRP1B, MUC16, PCDH15, PCLO, RYR2, SPTA1, TP53, TTN, XIRP2, ZFHX4, and other genes. Additionally, we found a large degree of amplification and deletion in the high-risk group. Finally, the IC_50_ value for multiple drugs was predicted to be significantly different between high- and low-risk groups based on the expression of all characteristic genes in the constructed model. In conclusion, our study provides new insights into the individualized treatment of LUAD.

The median risk score is used as a threshold to distinguish between high- and low-risk groups, and the essence of the grouping is to find factors influencing tumor development. Our study not only illustrates the differences between high- and low-risk scores in clinical traits and survival prognosis but also demonstrates the correlation between high- and low-risk groups in terms of genetic mutations, immune cell infiltration, and tumor drug resistance. The clinical term high risk includes mainly the risk of the disease, the risk of disease progression, the difficulty of curing the disease, and the risk of tumor recurrence. Thus, the high-risk group constructed in our study has an inclusive relationship with clinical high-risk, and our risk model can be used to predict the development of clinical risk and prognosis for survival, to assess the degree of clinical risk and to guide clinical treatment options.

There are still some limitations to our study. The problem of sample imbalance is an important and common problem in data analysis and is largely related to the original data itself and the method of analysis. Although we try to avoid the effects of sample imbalance when analyzing the data, the imbalance cannot be completely avoided, and we can only try to reduce the interference of sample imbalance in the analysis of data, the construction of models, and the evaluation of model validity. Although several independent external validations were performed in this study, there may be an innate case-selection bias in the results when collecting tissue and information retrospectively in publicly available databases. Therefore, reliable *in vitro* and *in vivo* experiments as well as large-scale prospective clinical trials are needed to confirm our findings.

In conclusion, we performed a comprehensive and systematic bioinformatics analysis and identified the TRP-related prognostic gene signature for LUAD patients. The TRP score is a promising biomarker for determining the prognosis, molecular subtype, tumor microenvironment, and drug selection in patients with LUAD. Therefore, our study provides new insights into the individualized treatment of LUAD.

## Data Availability

The original contributions presented in the study are included in the article/Supplementary Material; further inquiries can be directed to the corresponding author.

## Supplementary material

SUPPLEMENTARY TABLE S1 Chi-square test results of clinical characteristics in tumor group and control group.

SUPPLEMENTARY TABLE S2 Chi-square test results of the top 20 genes with the highest mutation frequency in the high-low risk group.

SUPPLEMENTARY TABLE S3 Chi-square test results of chromosome amplification and deletion in high and low-risk groups.
